# Supplementary material for: Mercury evidence for combustion of organic-rich sediments during the end-Triassic crisis
Source: Nat Commun. 2022 Mar 9;13:1307. doi: 10.1038/s41467-022-28891-8 (PMC8907283; doi:10.1038/s41467-022-28891-8)
Supplement: Supplementary file 1 — Supplementary Information [file 41467_2022_28891_MOESM1_ESM.pdf]

Description of Supplementary Files

File Name: Supplementary Information for **“Mercury evidence for combustion of organic-rich sediments during the end-Triassic crisis”** by Shen et al.

Description: Supplementary Notes (1-4), Supplementary Figures (1-2), and Supplementary References.

Supplementary Notes

Supplementary Note 1 | Mercury cycling in the ocean

Mercury exists in three main forms in the atmosphere[1]: (1) gaseous elemental Hg (Hg^0), (2) atmospheric Hg^{2+} compounds that are commonly associated with particles (Hg_p), and (3) halide compounds (referred to collectively as reactive gaseous mercury, RGM). Hg is distributed globally mainly in the form of Hg^0, which compromises 90% of total atmospheric Hg and has an atmospheric residence time of ~6 months to 1 year, allowing for long-distance transport[2]. Hg^0 can be oxidized to Hg^{2+}, which is removed from the atmosphere through both wet (RGM) and dry (Hg_p) deposition[2-6]. Hg^{2+} can be methylated into neurotoxic and bioaccumulative methyl mercury (MMHg) in the aqueous environment[4, 7], which poses a serious threat to human health via fish or rice consumption. In addition to atmospheric deposition, Hg is also delivered to the ocean by river-borne particles (e.g., clays and organic matter)[8].

In the aqueous environment, Hg is present mainly as elemental mercury (Hg^0_{aq}), divalent inorganic mercury (Hg^{2+}_{aq}), monomethyl mercury (MMHg), dimethyl mercury (DMHg), and particle-bound mercury (Hg^P_{aq})[9]. Hg has somewhat variable behavior with water depth in the ocean[10]. In the surface layer, Hg^{2+}_{aq} can be reduced to Hg^0_{aq} and then re-emitted to the atmosphere. Hg^{2+}_{aq} is also adsorbed onto suspended organic particulates (Hg^P_{aq}) in amounts that are generally proportional to primary production. Most Hg^P_{aq} is released back to the water column during remineralization of organic matter, leading to increased concentrations of Hg^{2+}_{aq} within the oceanic thermocline region. Methylation of Hg^{2+}_{aq} to MMHg and DMHg occurs mainly in oxygen-minimum zones (OMZs). Only a small fraction of Hg^P_{aq} reaches the deep-ocean floor to accumulate in abyssal deposits. In marine sediments, Hg tends to form strong, stable complexes with organic matter and/or HgS minerals and Hg-S complexes that resist remobilization in the burial environment[11, 12].

The large reservoir of Hg in the ocean (~953 × 10^6 mol) plays an important role in the Earth's Hg cycle[10]. In the open ocean, Hg concentrations are mainly between 0.1 and 0.2 pmol in the surface mixed layer, yielding an inventory of ~3 × 10^6 mol Hg[10]. The intermediate layer (~200–1000 m) often has the higher Hg concentrations (~0.4 pmol) due to remineralization of sinking organic matter, yielding an inventory of 110 × 10^6 mol Hg. Hg concentrations increase slightly downward to the

abyssal seafloor (~0.8 pmol), yielding an inventory in the deep ocean of 840×10^6 mol Hg. The residence times of Hg in the deep ocean (1700 yr), and intermediate layer (120 yr) are much longer than that in the surface mixed layer (7 months) and atmosphere (6–12 months)[10].

Supplementary Note 2 | Host phases of Hg in marine sediments

Robust use of Hg as a volcanic proxy requires an understanding of its sedimentary host phases. Most studies have assumed organic matter to be the dominant host of Hg, relying on mercury to total organic carbon ratios (Hg/TOC) to assess Hg enrichments in ancient sediments[12, 13], but Hg also can be associated with the sulfide[11, 12, 14] or clay fractions of the sediment[12, 15, 16]. The exact controls on Hg-host phase associations are not well understood, but uptake of Hg by sulfides appears to be linked to intensely reducing conditions that promote high stability constants for inorganic Hg-S complexes[11, 12, 14]. Clay minerals are also capable of adsorbing Hg, leading to significant Hg enrichments in some shale formations[12, 15].

The potential host phases of Hg can be proxied by total organic carbon (TOC; for the organic fraction), total sulfur (TS; for the sulfide fraction), and thorium (Th; for the clay fraction). The Katsuyama section has low contents of TOC (<0.2 %), TS (<0.1 %) and Th (mostly < 4 ppm), and Hg concentrations exhibit insignificant covariation with all three proxies ($r = +0.06, +0.20, +0.18$ for Hg-TOC, Hg-TS, and Hg-Th, respectively) (Supplementary Fig. 1). Thus, the dominant host phase of Hg in the present study samples remains uncertain. However, increases in both raw Hg and normalized Hg concentrations (i.e., Hg/TOC, Hg/TS, Hg/Al) around the Triassic–Jurassic extinction interval in the Katsuyama section support increased Hg loading from an external source (Fig. 2).

Supplementary Note 3 | Mercury isotopes as a proxy for Hg sources

Mercury has seven stable isotopes (196, 198, 199, 200, 201, 202 and 204) with relative mass variation of ~4 %[17]. Hg isotopes can be used to trace the sources and pathways of Hg in various environments[18]. Recent studies have discovered both mass-dependent fractionation (MDF) and mass-independent fractionation (MIF) of Hg isotopes in natural samples[17-19]. MDF results from differences in the zero-point energies of various isotopes, and can occur during various physical, chemical and biological processes[20]; it is not reported in this study. MIF is mainly caused by the

nuclear volume and magnetic isotope effects, and it can be used to infer specific processes such as Hg^0 volatilization, dark Hg(II) reduction, and photochemical reactions[17, 18]. Biotic and dark abiotic reactions do not produce significant MIF (i.e., Δ^{199}Hg). In contrast, all photochemical reactions that have been studied to date produce changes in MIF[17, 18]. The largest positive MIF of Hg isotopes is caused by photochemical degradation of methyl mercury in water, and the largest negative MIF of Hg isotopes is caused by photochemical reduction of inorganic Hg[18].

Mass-independent fractionation (MIF) of odd Hg isotopes (i.e., Δ^{199}Hg) can be used to identify certain processes (e.g., photochemical reduction) that influence Hg cycling (see review by Blum et al.[18]). Δ^{199}Hg values are near-zero for direct volcanic emissions from the deep Earth, distinguishing them from terrestrial and atmospheric fluxes, which generally show negative (median: -0.10 ‰, range: -0.28 ‰ to -0.02 ‰) and positive (median: 0.39 ‰, range: 0.09 ‰ to 0.97 ‰) Δ^{199}Hg values, respectively[18, 21] [note: all ranges are given as 25th-75th percentiles to avoid influnce by outliers]. Reservoir-specific MIF values can be used to interpret Hg sources, as has been done previously for sections of the Toarcian OAE[22], the Permian–Triassic boundary[16, 23, 24] and the T–J boundary[25, 26, 27].

In the present study, the slopes of linear relationships between Δ^{199}Hg and Δ^{201}Hg for the present (Katsuyama) and previous (Haojiagou, Qilixia, St. Audrie`s Bay, Nevada, and Levanto) settings are between 1.0 to 1.36 for both sections (Supplementary Fig. 2), which is consistent with values for photoreduction of aqueous Hg(II) driven by natural dissolved organic matter[18]. Below the T–J boundary, relatively positive Δ^{199}Hg values in deepwater (Katsuyama and Levanto) and negative Δ^{199}Hg values in terrestrial (Haojiagou and Qilixia) and nearshore (St. Audries Bay) settings document the dominant marine and continental sources of Hg, respectively (Fig. 4). This pattern is consistent with spatial variation of Hg isotopes in modern depositional systems. During the T–J transition, near-zero Δ^{199}Hg for all sections (Haojiagou, Qilixia St. Audrie`s Bay, Nevada, and Levanto) except Katsuyama may be the result of mainly volcanogenic Hg emissions and/or mixing of atmospheric Hg (positive) and terrestrial (negative) fluxes. The Katsuyama section exhibits unusually negative Δ^{199}Hg values around the T–J extinction interval, evidence that negative Δ^{199}Hg values source of Hg from atmosphere deposition.

Supplementary Note 4 | Wildfires records of the T–J transition

Widespread evidence of wildfire has been reported from T–J transitional sections at a global scale[28, 29]. The T–J extinction interval has yielded charcoal in Jameson Land Basin, East Greenland[30] ; pyrolytic polycyclic aromatic hydrocarbons (PAHs) in Germany and Sweden[31]; microscopic charcoal and combustion-derived pyrolytic PAHs in Poland[32]; charcoal abundance and reflectance in coal seams and coaly shales in Sweden and Denmark Danish Basin[33]; as well as pyrolytic PAHs in East Greenland[34], North China[35] and South China[36]. Therefore, the near-global distribution of wildfires is likely the product of a common origin associated with long-term climate change (warming and aridification) triggered by the CAMP eruptions.

Supplementary References

1. Schroeder, W. H. & Munthe, J. Atmospheric mercury—an overview. *Atmospheric Environ.* **32**, 809-822 (1998).
2. Selin, N. E. Global biogeochemical cycling of mercury: a review. *Annu. Rev. Environ. Resour.* **34**, 43-63 (2009).
3. Mason, R. P., Fitzgerald, W. F., Morel, F. M. The biogeochemical cycling of elemental mercury: anthropogenic influences. *Geochim. Cosmochim. Ac.* **58**, 3191-3198 (1994).
4. Lamborg, C. H., Fitzgerald, W. F., O'Donnell. J., Torgersen, T. A non-steady-state compartmental model of global-scale mercury biogeochemistry with interhemispheric atmospheric gradients. *Geochim. Cosmochim. Ac.* **66**, 1105-1118 (2002).
5. Clarkson, T. W. Mercury: major issues in environmental health. *Environ. Health Perspect.* **100**, 31-38 (1992).
6. Feng, X. & Qiu, G. Mercury pollution in Guizhou, Southwestern China—an overview. *Sci. Total Environ.* **400**, 227-237 (2008).
7. Amyot, M., Gill, G. A., Morel, F. M. Production and loss of dissolved gaseous mercury in coastal seawater. *Environ. Sci. Technol.* **31**, 3606-3611 (1997).
8. Liu, M., Zhang, Q., Maavara, T., Liu, S., Wang, X. & Raymond, P. A. Rivers as the largest source of mercury to coastal oceans worldwide. *Nat. Geosci.* **14**, 672-677 (2021).
9. Mason, R. P. & Fitzgerald, W. F. The distribution and biogeochemical cycling of mercury in the equatorial Pacific Ocean. *Deep Sea Res. Pt. I* **40**, 1897-1924 (1993).

10. Zhang, Y., Jaeglé, L., Thompson, L. Natural biogeochemical cycle of mercury in a global three-dimensional ocean tracer model. *Global Biogeochem. Cy.* **28**, 553-570 (2014).

11. Shen, J. et al. Mercury in marine Ordovician/Silurian boundary sections of South China is sulfide-hosted and non-volcanic in origin. *Earth Planet. Sci. Lett.* **511**, 130-140 (2019).

12. Shen, J. et al. Sedimentary host phases of mercury (Hg) and implications for use of Hg as a volcanic proxy. *Earth Planet. Sci. Lett.* **543**, 116333 (2020).

13. Ravichandran, M. Interactions between mercury and dissolved organic matter—a review. *Chemosphere* **55**, 319-331 (2004).

14. Bower, J., Savage, K. S., Weinman, B., Barnett, M. O., Hamilton, W. P. & Harper, W. F. Immobilization of mercury by pyrite (FeS_2). *Environ. Pollut.* **156**, 504-514 (2008).

15. Farrah, H. & Pickering, W. F. The sorption of mercury species by clay minerals. *Water Air Soil Poll.* **9**, 23-31 (1978).

16. Shen, J. et al. Mercury evidence of intense volcanic effects on land during the Permian–Triassic transition. *Geology* **47**, 1117-1121 (2019).

17. Bergquist, B. A. & Blum, J. D. Mass-dependent and-independent fractionation of Hg isotopes by photoreduction in aquatic systems. *Science* **318**, 417-420 (2007).

18. Blum, J. D., Sherman, L. S. & Johnson, M. W. Mercury isotopes in earth and environmental sciences. *Annu. Rev. Earth Planet. Sci.* **42**, 249-269 (2014).

19. Chen, J., Hintelmann, H., Feng, X. & Dimock, B. Unusual fractionation of both odd and even mercury isotopes in precipitation from Peterborough, ON, Canada. *Geochim. Cosmochim. Ac.* **90**, 33-46 (2012).

20. Perrot, V., Bridou, R., Pedrero, Z., Guyoneaud, R., Monperrus, M., Amouroux, D. Identical Hg isotope mass dependent fractionation signature during methylation by sulfate-reducing bacteria in sulfate and sulfate-free environment. *Environ. Sci. Technol.* **49**, 1365-1373 (2015).

21. Yin, R. et al. Mercury isotopes as proxies to Identify sources and environmental impacts of mercury in Sphalerites. *Sci. Rep.* **6**, 18686 (2016).

22. Them II, T. et al. Terrestrial sources as the primary delivery mechanism of mercury to the oceans across the Toarcian Oceanic Anoxic Event (Early Jurassic). *Earth Planet. Sci. Lett.* **507**, 62-72 (2019).

23. Grasby, S. E. et al. Isotopic signatures of mercury contamination in latest Permian oceans.

Geology **45**, 55-58 (2017).

24. Shen, J. et al. Evidence for a prolonged Permian–Triassic extinction interval from global marine mercury records. *Nat. Commun.* **10**, 1563 (2019).
25. Thibodeau, A. M. et al. Mercury anomalies and the timing of biotic recovery following the end-Triassic mass extinction. *Nat. Commun.* **7**, 11147 (2016).
26. Yager, J. A. et al. Mercury contents and isotope ratios from diverse depositional environments across the Triassic–Jurassic Boundary: Towards a more robust mercury proxy for large igneous province magmatism. *Earth-Sci. Rev.* **223**, 103775 (2021).
27. Shen, J. et al. Intensified continental chemical weathering and carbon-cycle perturbations linked to volcanism during the Triassic–Jurassic transition. *Nat. Commun.* **13**, 299 (2022).
28. Lindström, S. et al. Tracing volcanic emissions from the Central Atlantic Magmatic Province in the sedimentary record. *Earth-Sci. Rev.* **212**, 103444 (2021).
29. Pieńkowski, G., Niedźwiedzki, G., Waksmundzka, M. Sedimentological, palynological and geochemical studies of the terrestrial Triassic–Jurassic boundary in northwestern Poland. *Geol. Mag.* **149**, 308-332 (2012).
30. Belcher, C. M. et al. Increased fire activity at the Triassic/Jurassic boundary in Greenland due to climate-driven floral change. *Nat. Geosci.* **3**, 426-429 (2010).
31. Van de Schootbrugge, B. et al. Floral changes across the Triassic/Jurassic boundary linked to flood basalt volcanism. *Nat. Geosci.* **2**, 589-594 (2009).
32. Marynowski, L. & Simoneit, B. R. Widespread Upper Triassic to Lower Jurassic wildfire records from Poland: evidence from charcoal and pyrolytic polycyclic aromatic hydrocarbons. *Palaios* **24**, 785-798 (2009).
33. Petersen, H. I. & Lindström, S. Synchronous wildfire activity rise and mire deforestation at the Triassic–Jurassic boundary. *PLoS One* **7**, e47236 (2012).
34. Williford, K. H., Grice, K., Holman, A., McElwain, J.C. An organic record of terrestrial ecosystem collapse and recovery at the Triassic–Jurassic boundary in East Greenland. *Geochim. Cosmochim. Ac.* **127**, 251-263 (2014).
35. Zhang, X. et al. Wildfire records across the Triassic–Jurassic Boundary in the Southern margin of the Junggar Basin. *Acta Sedimentol. Sin.* (2021). Doi: 10.14027/j.issn.1000-0550.2020.103. (In Chinese with English abstract)

36. Song, Y. et al. Distribution of pyrolytic PAHs across the Triassic-Jurassic boundary in the Sichuan Basin, southwestern China: Evidence of wildfire outside the Central Atlantic Magmatic Province. *Earth-Sci. Rev.* **201**, 102970 (2020).

Supplementary Figures:

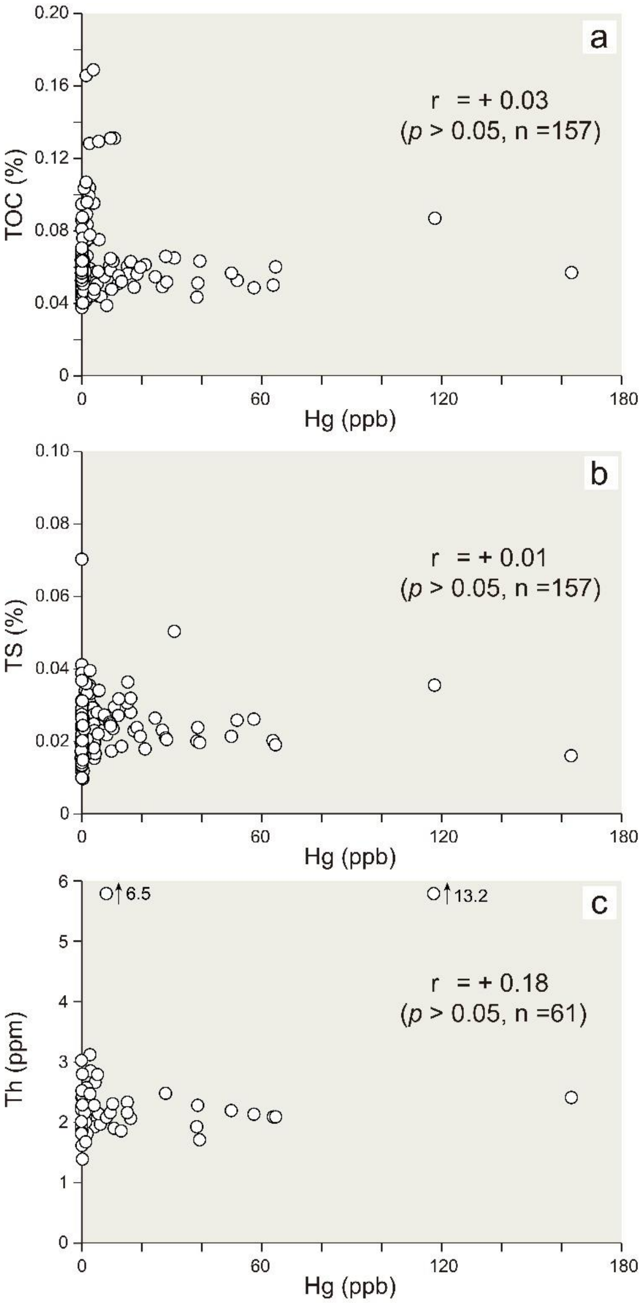

Supplementary Figure 1. Crossplots of total organic carbon (TOC, %) vs mercury (Hg, ppb) (a), total sulfur (TS, %) vs Hg (ppb) (b), and thorium (Th, ppm) vs Hg (ppb) (c) for the Katsuyama section. Bracketed numbers are the number of analyzed samples, and *r* is the correlation coefficient.

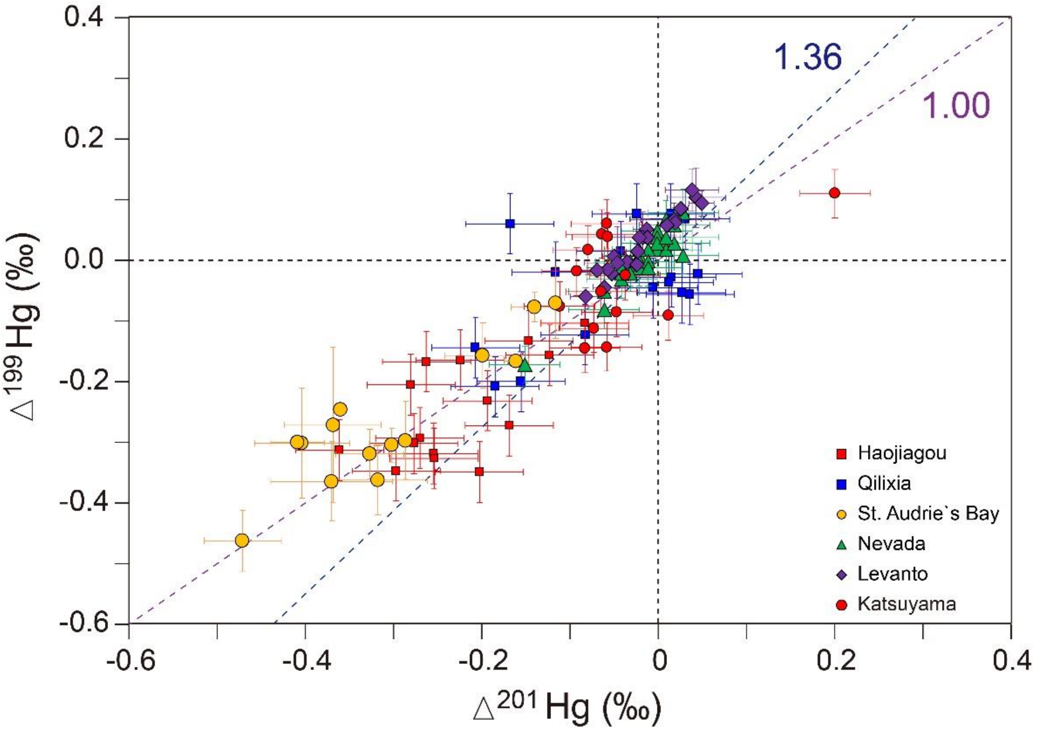

Supplementary Figure 2. Crossplots of Δ^{199}Hg versus Δ^{201}Hg for all the sections near the Triassic–Jurassic transition. The solid line indicates a slope of 1.00, the dashed line a slope of 1.20, and the dotted line a slope of 1.36 (from reference[18]). The horizontal and vertical bars represent standard deviation (2σ) values for Δ^{201}Hg and Δ^{199}Hg, respectively. Data sources for the sections: Katsuyama (this study); Levanto[26]; Nevada[25]; St. Audrie`s Bay[26], as well as Haojiagou and Qilixa[27].
