# Supplementary material for: Mercury evidence for combustion of organic-rich sediments during the end-Triassic crisis
Source: Nat Commun. 2022 Mar 9;13:1307. doi: 10.1038/s41467-022-28891-8 (PMC8907283; doi:10.1038/s41467-022-28891-8)
Supplement: Supplementary file 2 — Description of Additional Supplementary Files [file 41467_2022_28891_MOESM2_ESM.pdf]

Description of Additional Supplementary Files

Supplementary Data 1: Geochemical dataset for the study section.
